# Against All Odds? Addiction History Associated with Better Viral Hepatitis Care: A Dutch Nationwide Claims Data Study

**DOI:** 10.3390/jcm11041146

**Published:** 2022-02-21

**Authors:** Daan W. Von den Hoff, Floor A. C. Berden, Femke Atsma, Arnt F. A. Schellekens, Joost P. H. Drenth

**Affiliations:** 1 Department of Gastroenterology and Hepatology, Radboud University Medical Center, 6525 GA Nijmegen, The Netherlands; floor.berden@radboudumc.nl; 2 Scientific Center for Quality of Healthcare (IQ Healthcare), Radboud Institute for Health Sciences, Radboud University Medical Center, 6500 HB Nijmegen, The Netherlands; femke.atsma@radboudumc.nl; 3 Department of Psychiatry, Radboud University Medical Centre, 6525 GA Nijmegen, The Netherlands; arnt.schellekens@radboudumc.nl; 4 Nijmegen Institute for Scientist-Practitioners in Addiction (NISPA), Radboud University, 6500 HE Nijmegen, The Netherlands; 5 Donders Institute for Brain, Cognition and Behavior, Radboud University, 6525 AJ Nijmegen, The Netherlands

**Keywords:** hepatitis B, hepatitis C, substance-related disorders/epidemiology (MeSH), healthcare utilization, elimination

## Abstract

The elimination of viral hepatitis in target populations is crucial in reaching WHO viral hepatitis elimination goals. Several barriers for the treatment of viral hepatitis in people with addictive disorders have been identified, yet nationwide data on hepatitis healthcare utilization (HCU) in these patients are limited. We investigated whether a history of addictive disorder is associated with suboptimal hepatitis HCU, indicating failure to receive diagnostic care or treatment. We identified all newly referred viral hepatitis patients in the Netherlands between 2014 and 2019 by query of the Dutch national hospital claims database. Each patient’s first year of HBV or HCV care activities was collected and clustered in two categories, ‘optimal’ or ‘suboptimal’ hepatitis HCU. Optimal HCU includes antiviral therapy. We tested the association between addiction history and HCU, adjusted for sex, age, migrant status, and comorbidity. In secondary analyses, we explored additional factors affecting hepatitis HCU. We included 10,513 incident HBV and HCV patients, with 13% having an addiction history. Only 47% of all patients achieved optimal hepatitis HCU. Addiction history was associated with less suboptimal HCU (adjusted OR = 0.73, 95% CI = 0.64–0.82). Migration background was associated with suboptimal HCU (OR = 1.62, 95% CI = 1.50–1.76). This study shows that addiction history is associated with higher viral hepatitis HCU; thus, this population performs better compared to non-addicted patients. However, less than 50% of all patients received optimal hepatitis care. This study highlights the need to improve hepatitis HCU in all patients, with a focus on migrant populations. Linkage to care in the addicted patients is not studied here and may be a remaining obstacle to be studied and improved to reach WHO viral hepatitis elimination goals.

## 1. Introduction

Patients with a chronic hepatitis infection are at risk of developing severe long-term liver-related complications including liver cirrhosis and hepatocellular carcinoma. In recent years, both hepatitis B (HBV) and C (HCV) virus infections have become manageable with drugs that suppress the hepatitis B virus and even cure patients in the case of HCV [1,2]. The worldwide prevalence of chronic HBV and HCV is around 3.9% and 1.0%, respectively [3]. In most Western countries, the prevalence of viral hepatitis is lower, and in the Netherlands, it is estimated to be around 0.3% [4]. In 2016, the World Health Organization (WHO) set ambitious goals for the elimination of HBV and HCV by 2030 [5]. Key to the target in this elimination strategy are specific populations, where the prevalence rates are higher, in particular migrants from endemic countries and people who inject(ed) drugs (PWID) [4,6]. For instance, in the Netherlands prevalence rates for HBV and HCV among PWID are up to 4% and 59%, respectively [4]. Treating this population, as part of a micro-elimination strategy, is crucial to reach WHO goals [7].

HBV and HCV elimination in PWID is challenging, as studies among PWID show mediocre rates of treatment initiation, varying between 20−90% [8,9,10,11,12,13,14,15]. Failure to initiate treatment puts patients at a risk of liver-related complications and maintains a viral reservoir. Several reasons have been put forward to explain low treatment rates in PWID, such as absence of insurance coverage [16], logistic inability to utilize care services outside the patient’s community [17], and personal barriers for engaging in testing and treatment [18,19,20]. Treating physicians might also experience barriers for treatment initiation in PWID, as they may deem them incapable of adhering to treatment [19,21,22]. The ultimate fallout might be that PWID with chronic HBV or HCV underutilize hospital care and thus remain infected. To our knowledge, no data on PWID HBV and HCV healthcare utilization (HCU) are yet available.

Dutch HBV and HCV care is primarily provided in hospitals, so utilization of hospital diagnostic and treatment services is essential for the elimination of HBV and HCV. The aim of this study was to compare the HBV and HCV-related healthcare utilization (HCU) in patients with an addiction history (AH+) versus the non-addicted population (AH−) using a national healthcare claims database in the Netherlands. We opted for AH as the variable of interest, while potential addiction-associated barriers to participate in hospital care determine HCU in PWID, not injecting drug use per se. Moreover, in the Dutch viral hepatitis population, injecting drug use is the most likely cause of infection when addiction is present [23]. We hypothesized that a history of addiction is associated with suboptimal HCU.

## 2. Materials and Methods

### 2.1. Data Source

We used hospital claims data in the Netherlands from the years 2012–2019 as provided by Statistics Netherlands (CBS) [24]. This database consisted of anonymized diagnoses and treatment codes (‘DBC’) and all underlying diagnosis and treatment-related healthcare activities in hospital and addiction care. The database has a 99% coverage of the Dutch population, varying by year of submission 92.8–99.9% [25,26].

Diagnoses dated from 1 Januray 2012 up to 31 December 2019 were available in this database. In addition, age, sex, country of birth, and medication prescriptions were also available. In the Netherlands, addiction care is widely available and generally provided in low threshold, dedicated addiction care facilities, focusing on prevention and harm reduction [27,28]. They are a part of insured mental healthcare. Claims and diagnosis labels from these facilities were available in the database as well. Addiction history (AH+/−) was collected from these mental healthcare claims data. 

### 2.2. Study Population 

We selected all insured patients with available HBV or HCV diagnoses in Dutch hospitals from 2012 up to and including 2019. Hepatitis treatment was not performed by general practitioners or other primary care physicians in the timeframe this database covers. To identify a new referral, we excluded patients with an HBV/HCV diagnosis prior to 1 January 2014. Patients with an incident diagnosis after 1 January 2019 were excluded to ensure one year of follow-up, and patients could not be included twice (reinfection). The date of the first claim with an HBV or HCV diagnosis label was defined as the index date. Demographic data for all included patients were collected. Migrant status was defined as not born in the Netherlands. This database contains only documented migrant populations, these patients have full Dutch health care coverage [25]. Comorbid diagnoses, based on hospital care diagnosis labels, were organized according to the Charlson comorbidity index [29]. No distinction between HBV and HCV could be made based on diagnosis label, so we used HBV/HCV specific medication and ICD10 codes if available in order to characterize the type of infection. All individuals with at least one DBC claim from an addiction care center were allocated to the ‘AH+’ subgroup. Patients under 18 years old at the time of referral were excluded.

### 2.3. Outcomes and Clustering Healthcare Activities

The primary outcome is the odds of suboptimal viral hepatitis-related HCU in the first year after the index date for patients with and without AH. To describe and compare hepatitis HCU, all registered healthcare activities within HBV or HCV claims were aggregated to representative categories. Based on HBV and HCV guidelines [1,2,25,26], we constructed HCU stages with incremental adherence to the most optimal diagnostic and treatment trajectory. This was performed in collaboration with a panel of Dutch hepatitis experts. We presented the stages to the panel, which resulted in a hybrid review process. Agreement was reached in the form of our initial proposal, and it was not adjusted during review. The final outcome determined the conclusive definition for the endpoint. We distinguished optimal hepatitis HCU and suboptimal HCU. Suboptimal care was further divided into grade I (mild) and grade II (severe) suboptimal care for our subgroup analysis. 

Patients in the ‘optimal care’ HCU category were defined as having visited the outpatient clinic more than twice and/or received laboratory tests more than twice and/or underwent abdominal radiology (i.e. sonography) or biopsy (to assess liver fibrosis or cirrhosis) and had been prescribed HBV or HCV medication. Patients with ‘grade I suboptimal care’ were defined as having received the same diagnostic care as the optimal group but did not initiate treatment. ‘Grade II suboptimal care’ was defined as loss to follow-up early in the diagnostic chain (maximum of two outpatient visits and two laboratory tests and no other care). 

We anticipated that physicians may have stalled treatment initiation in the years prior to direct-acting antiviral (DAA) availability and reimbursement in late 2015. To adjust for this potential confounding effect, we included all medication prescriptions up to 31 December 2016 in patients diagnosed before 1 January 2016. A detailed description of all categories of HCU can be found in Supplement Table S1. 

### 2.4. Statistical Analyses

Baseline characteristics were summarized as percentages, means (with SD), or medians (IQR), depending on the variable and its distribution. Abdominal radiology, other interventions, and pathologic assessment were summarized as percentage of patients that underwent at least one activity in the subgroup. Odds for suboptimal HCU were compared between patients with and without a history of addiction treatment, using binary logistic regression analyses, while taking age, sex, migrant status, and comorbid conditions (especially HIV) into account. First, we tested the association between addiction history and suboptimal HCU. Second, we tested the association between addiction history and grade II suboptimal HCU in the subgroup of patients who received suboptimal care. A sensitivity analysis was performed by substituting addiction history (AH+) with a history of opioid use disorder as determinant. Since no data on injecting drugs were available in the CBS national claims database, and the most commonly injected drug in the Netherlands is opioids, a history of opioid use disorder might best describe the PWID population within viral hepatitis patients. This sensitivity analysis adds external validity to our results, as in literature the determinant is often PWID [1,2,4,20,30,31,32]. 

We explored additional factors influencing HCU in a secondary analysis, using backward stepwise regression analysis. Outcomes are presented as odds ratios. All analyses were performed using Statistical Package for the Social Sciences version 25.

## 3. Results

### 3.1. Study Population

**Figure 1:**
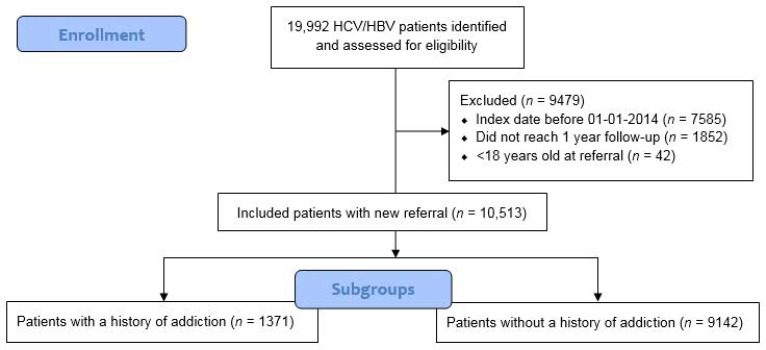
Patient flowchart.

We identified 19,992 patients with an HBV or HCV diagnosis from 2012 to 2019 in our database. The number of new diagnoses remained relatively stable at around 2100 (range 1933–2430) per year (Supplement Table S2). After exclusion, 10,513 newly referred patients were included (Figure 1). The majority of patients was male (62%) and mean age was 47.5 years old (SD: 13.6). Some 13% (*n* = 1371) of the included patients had a history in addiction care (AH+). Demographic data and comorbid conditions are shown in Table 1. The majority of viral hepatitis patients had a migration background (63.1%), with more migrants in the AH− hepatitis population than in the AH+ patients (68.0% vs. 32.2%). Patients with a migration background mainly originated from Africa or Asia (64.2%) and Eastern Europe (15.3%). Malignant disease was the most prevalent registered comorbid condition in our viral hepatitis population (6.4%), followed by HIV/AIDS (5.3%). Liver cirrhosis was more prevalent in AH+ patients than in AH− patients (4.7% vs. 2.4%). Although a distinction could not be made in 30% of patients, our data suggest that HCV and HBV distribution were different in AH+ patients compared to AH− patients, with more HCV in AH+ (72% vs. 34%) and more HBV in AH− (32% vs. 4%) (Table 1).

### 3.2. Healthcare Utilization (HCU)

All aggregated healthcare activity categories showed a skewed distribution (Table 2). AH+ patients had a median of four outpatient visits compared to three in AH− patients. Both subgroups underwent a median of two venipunctures. Abdominal radiology (67.5% vs. 63.4%) and other interventions, i.e. endoscopic or surgical procedures (13.6% vs. 8.9%), were more frequently seen in AH+ patients.

**Figure 2:**
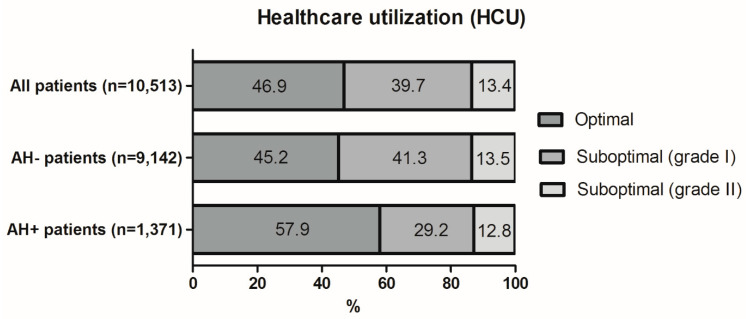
Viral hepatitis-related healthcare utilization.

We found that AH+ patients more often received optimal care than AH− patients (57.9% vs. 45.2%) (Figure 2 and Supplement Table S3). Binary logistic regression analysis demonstrated lower odds for suboptimal HCU in AH+ patients compared with AH− patients after adjusting for other variables (aOR = 0.73, 95% CI = 0.64–0.82) (Table 3). In the subgroup of patients with suboptimal HCU, addiction history had an unadjusted association with grade II suboptimal care (OR = 1.35, 95% CI = 1.12–1.63); however, this effect disappeared after adjustment for potential confounding variables (aOR = 1.13, 95% CI = 0.93, 1.38; Table 3). The sensitivity analysis with history of opioid use disorder instead of addictive disorder in general did not alter the results (Supplement Table S4). 

### 3.3. Exploratory Model

**Figure 3:**
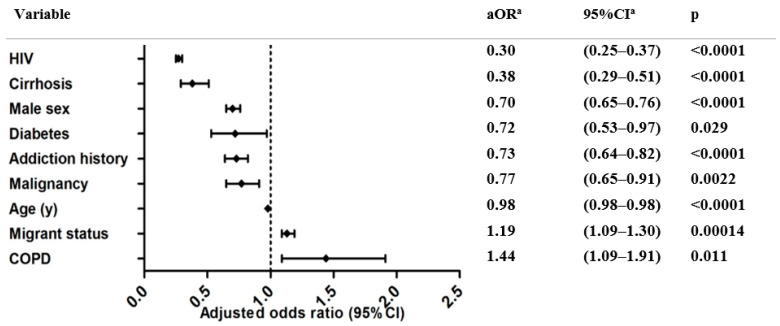
Identification of factors contributing to suboptimal (I + II) hepatitis HCU. ^a^: OR = odds ratio; CI = confidence interval; aOR = adjusted odds ratio; adjusted for sex, age, migrant status, cirrhosis, diabetes, COPD, malignancy, HIV. Optimal = diagnosed and treated; suboptimal I = diagnosed and followed-up; suboptimal II = only diagnosed.

Our exploratory logistic regression model, testing other factors for an association with suboptimal hepatitis HCU, showed that migration background increased the odds for suboptimal HCU (OR = 1.19, 95% CI = 1.09–1.30). Multivariate analysis also identified COPD to be associated with suboptimal HCU (aOR = 1.44, 95% CI = 1.09–1.91) (Figure 3). All other factors decreased the odds of suboptimal HCU (Figure 3 and Supplement Table S5A).

**Figure 4:**
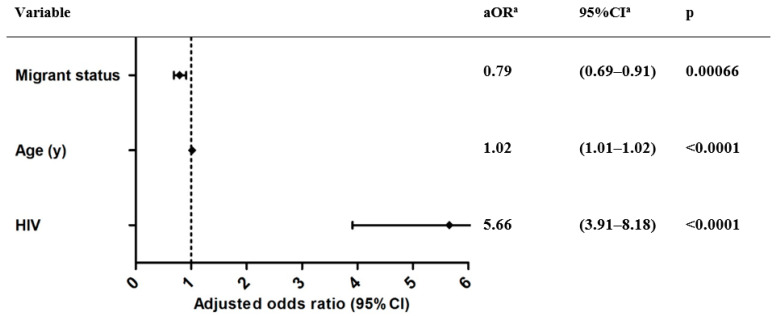
Identification of factors contributing to grade II suboptimal hepatitis HCU. ^a^: OR = odds ratio; CI = confidence interval; aOR = adjusted odds ratio; adjusted for sex, age, migrant status, cirrhosis, diabetes, COPD, malignancy, HIV. Optimal = diagnosed and treated; suboptimal I = diagnosed and followed-up; suboptimal II = only diagnosed.

When exploring patient characteristics associated with grade II suboptimal hepatitis HCU as opposed to grade I suboptimal HCU, migration background decreased the odds for grade II suboptimal HCU (OR = 0.64, 95% CI = 0.57–0.73), while age and HIV status increased the odds of grade II suboptimal HCU (Supplement Table S5B) (Figure 4).

## 4. Discussion

This study shows that HCU in the Dutch patients with an addiction history is better compared to those without a history of addiction. Our exploratory analysis suggests that, among referred viral hepatitis patients, a migration background is associated with suboptimal hepatitis HCU. Overall, optimal viral hepatitis HCU in the first year after referral was low at 47%, so increased efforts to achieve optimal care in all viral hepatitis patients is warranted.

Our findings indicate that enrolment in Dutch addiction care for addiction to any substance predicts better HCU for viral hepatitis compared to those without a history of addiction. The association between addiction and optimal HCU contrasts with our hypothesis and existing literature linking PWID with poor HBV and HCV care outcomes. In the literature, PWID are found to perform worse in treatment initiation for viral hepatitis [8,10,12,22]. There are a few possible explanations for this discrepancy. Most importantly, in the Netherlands, addiction care is provided through specialized addiction care facilities, and it is insured and widely available [27,28]. These facilities provide needle exchange programs, access to opioid substitution therapy with methadone, buprenorphine, and heroin-assisted treatment [23,33]. These interventions facilitate low threshold monitoring and education of the addicted population on topics such as viral hepatitis, potentially contributing to more optimal hepatitis HCU. Addiction might influence HCU more in countries without the aforementioned interventions, and policymakers should therefore consider implementing such programs. Second, this study did not include patients who were never referred for hepatitis care and for whom a hospital claim is therefore absent. Although not studied here, a previous study has identified this linkage-to-care problem in Dutch addiction care centers [7]. What we can conclude, however, is that there is no reason to withhold viral hepatitis patients with comorbid addiction from referral for hepatitis care and that stigmatization toward the addicted population in terms of hepatitis HCU is unjustified and should be avoided at all times. Reduced stigmatization and efforts to increase linkage to care will result in higher treatment initiation and completion rates for the addicted population, further approaching the WHO goals of viral elimination.

Nearly two-thirds of the viral hepatitis population in our database had a migration background. The association between migration background and suboptimal HCU found in our exploratory analysis is the major contributor to the observed low overall rate of optimal HCU (47%). Other contributors may be HBV patients without an indication for treatment or HCV patients who cleared the infection spontaneously; both are presumably present in a limited number of patients. The migrant population might fail to achieve optimal HCU due to a mix of barriers for hepatitis HCU, such as language and insurance issues [34,35]. Interestingly, we found that within the suboptimal HCU group, migrant status increased the odds of grade I suboptimal HCU and not grade II suboptimal HCU, indexed by early drop-out during the diagnostic trajectory. This suggests that patients with a migration background receive adequate diagnostic care but fail to initiate treatment. This finding calls for a need to better facilitate viral hepatitis treatment for this population. The opposite HCU effect was found in HIV-positive patients, as HIV status was strongly associated with optimal hepatitis HCU (OR: 3.33). However, when only patients with suboptimal HCU were analyzed, HIV was associated with grade II suboptimal HCU and thus loss to follow-up (OR: 5.9). This could be explained by the structured combined HIV and viral hepatitis care in Dutch HIV care facilities, contributing to more optimal hepatitis HCU. At the same time, if HIV-positive patients withdraw from healthcare, they seem to completely stop visiting the hospital, dropping out of viral hepatitis care. Besides migration background, COPD was also associated with suboptimal HCU. Only 2% of patients had a COPD medical history in the hospital, and many patients may be under COPD care in the non-hospital setting, so the relevance and generalizability of this finding are unknown. 

This study’s key strength is its national coverage, resulting in a large sample size and representative sample. Since the Netherlands has a relatively low number (0.145%) of uninsured persons, our study population in fact reflects the actual population [36]. The source data themselves are reliable, as they contain a centralized extensive overview of Dutch hospital care, as well as detailed information on addiction care claims. Furthermore, for our primary outcome, we performed logistic regression to take potential confounding effects of other factors into account. Lastly, we performed an explorative analysis to complement our findings and to provide guidance for future research and public health policymakers. 

This study also comes with limitations. Our outcomes are based on a variety of healthcare activity data, which we aggregated and constructed into representative stages of HCU (optimal, grade I suboptimal, grade II suboptimal). Though these categories were established in close collaboration with a panel of hepatitis treatment experts, increasing validity, it may not apply to other countries with different data registries. This method may be hard to replicate in other countries with different data registries. Secondly, there was no option to isolate PWID in our database and specifically test for an association between injecting drugs and hepatitis HCU. Though our sensitivity analysis, only testing an association among viral hepatitis patients with a history of opioid use disorder, did not alter the results, future studies may specifically look at PWID instead of addiction history. Another important limitation is the absence of a distinction between HCV and HBV infection in our database. This could be relevant, as HBV patients without an indication for treatment or HCV patients who cleared the infection spontaneously will have lowered our ‘optimal care’ outcome. Lastly, linkage to care is an important topic in viral hepatitis care for the addicted population and was not studied here, as primary care data were unavailable. As mentioned before, we believe the linkage-to-care issue may be substantial in the Dutch addicted population [7]. The most recent estimate of the Dutch HBV and HCV PWID population size in 2016 is 3640 (low–high estimate 2152–6056), while we retrieved 1371 patients with an addiction history from our national hospital database [4]. 

To summarize, in contrast to our hypothesis, a history of an addictive disorder is associated with less suboptimal hepatitis HCU in patients initiating hepatitis B and C care in the Netherlands. However, it must be noted that, overall, less than half of all patients initiating care for viral hepatitis received optimal hepatitis care. A migrant background was the most relevant factor contributing to suboptimal HCU. To reach WHO goals of viral elimination in the Netherlands, we recommend endeavors to increase hepatitis HCU, most importantly treatment initiation in all viral hepatitis patients, with extra focus on populations with a migration background. Efforts to eliminate HBV and HCV among PWID should focus on linkage to care, as we found that once patients with a history of addictive disorders are linked to care, they perform relatively well in terms of HCU. Stigma towards this population in terms of healthcare utilization is therefore unjustified. In countries without the availability of low threshold, insured addiction care, development of these services might provide an important vehicle for reaching WHO viral elimination for PWID. 

## Figures and Tables

**Table 1:** Baseline characteristics**.**

	Total *n* (%)	AH+ *n* (%)	AH− *n* (%)
Total	10,513	1371 (13.0)	9142 (87.0)
Male	6521 (62.0)	1090 (79.5)	5431 (59.4)
Age at diagnosis (y) (SD)	47.49 (13.6)	50.48 (9.0)	47.04 (14.1)
Comorbid conditions (any):	2190 (20.8)	360 (26.3)	1830 (20.0)
- Liver cirrhosis	282 (2.7)	65 (4.7)	217 (2.4)
- Malignancy	677 (6.4)	86 (6.3)	591 (6.5)
- HIV/AIDS	552 (5.3)	85 (6.2)	467 (5.1)
- Chronic obstructive pulmonary disease (COPD)	217 (2.1)	95 (6.9)	122 (1.3)
- Diabetes	198 (1.9)	17 (1.2)	181 (2.0)
Migrant status: *	6636 (63.1)	441 (32.2)	6195 (68.0)
- Western Europe	235 (3.5)	59 (13.4)	176 (2.8)
- Northern Europe	132 (2.0)	26 (5.9)	106 (1.7)
- Southern Europe	273 (4.1)	52 (11.8)	221 (3.6)
- Eastern Europe	1014 (15.3)	88 (20.0)	926 (14.9)
- Africa	1656 (25.0)	62 (14.1)	1594 (25.7)
- Asia	2603 (39.2)	65 (14.7)	2538 (41.0)
- Other **	723 (10.9)	89 (20.2)	634 (10.2)
Type of viral hepatitis			
- HCV	4105 (39.0)	992 (72.4)	3113 (34.1)
- HBV	2978 (28.3)	56 (4.1)	2922 (32.0)
- HCV + HBV co-infection	285 (2.7)	28 (2.0)	257 (2.8)
- Unknown	3145 (29.9)	295 (21.5)	2850 (31.2)

* 28 patients’ country of birth was missing**;** ** North America, South America, and Oceania; SD = standard deviation; AH = addiction history.

**Table 2:** Healthcare activities per category.

Category	Total Median (IQR)	AH+ Median (IQR)	AH− Median (IQR)
Outpatient visits	3 (2–6)	4 (2–6)	3 (2–6)
Venipunctures	2 (1–4)	2 (1–4)	2 (1–4)
Lab tests	19 (4–42)	24 (6–46)	19 (4–41)
Radiology(abdominal) *	63.9%	67.5%	63.4%
Other interventions (i.e., endoscopy) *	9.5%	13.6%	8.9%
Pathology *	3.2%	3.0%	3.2%

* % of patients that underwent at least 1 activity; IQR = interquartile range.

**Table 3:** **A**: Association between history of an addictive disorder and suboptimal (I + II) healthcare utilization. **B**: Association between history of an addictive disorder and grade II suboptimal healthcare utilization as opposed to grade I suboptimal HCU.

**(A)**						
**Factor**	**OR ^a^** **(Univariate)**	**95% CI ^a^**	***p***	**aOR ^ab^** **(Multivariate)**	**95% CI**	***p***
Addiction history	0.60	(0.54, 0.67)	<0.0001	0.73	(0.64–0.82)	<0.0001
**(B)**						
**Factor**	**OR ^a^** **(Univariate)**	**95% CI ^a^**	***p***	**aOR ^ab^** **(Multivariate)**	**95% CI**	***p***
Addiction history	1.35	(1.12, 1.63)	0.0020	1.15	(0.94–1.40)	0.17

^a^ OR = odds ratio; CI = confidence interval; aOR = adjusted odds ratio. ^b^ Adjusted for sex, age, migrant status, cirrhosis, diabetes, COPD, malignancy, HIV. Optimal = diagnosed and treated; suboptimal I = diagnosed and followed-up; suboptimal II = only diagnosed.

## Data Availability

Results are based on calculations by the authors using non-public microdata from Statistics Netherlands. These microdata are accessible for statistical and scientific research. For further information: microdata@cbs.nl.

## Supplementary Materials

The following supporting information can be downloaded at: https://www.mdpi.com/article/10.3390/jcm11041146/s1, Supplement Table S1: required number of healthcare activities for healthcare utilization (HCU) outcomes; Supplement Table S2: Year of diagnosis; Supplement Table S3: HCV/HBV healthcare utilization (HCU); Supplement Table S4. A: Sensitivity analysis. Association between opioid addiction and suboptimal healthcare utilization; B: Sensitivity analysis. Association between opioid addiction and grade II suboptimal healthcare utilization within suboptimal HCU subgroup; Supplement Table S5. A: Identification of variables contributing to HCU. Association between variables and suboptimal healthcare utilization; B: Identification of variables contributing to HCU. Association between variables and grade II suboptimal healthcare utilization within suboptimal HCU subgroup.
