# Supplementary material for: Against All Odds? Addiction History Associated with Better Viral Hepatitis Care: A Dutch Nationwide Claims Data Study
Source: J Clin Med. 2022 Feb 21;11(4):1146. doi: 10.3390/jcm11041146 (PMC8878485; doi:10.3390/jcm11041146)
Supplement: Supplementary file 1 [file jcm-11-01146-s001.zip › jcm-1560788-supplementary.pdf]

SUPPLEMENTARY

Table S1: Required number of healthcare activities for healthcare utilization (HCU) outcomes**.**

STAGE OF HCU	SEVERE SUBOPTIMAL	MILD SUBOPTIMAL	OPTIMAL
OUTPATIENT VISITS	≤2	>2	>2
	AND	*OR*	*OR*
BLOOD WITHDRAWAL	≤2	>2	>2
	AND	*OR*	*OR*
INTERVENTIONS	NO	≥1	≥1
IE. ABDOMINAL RADIOLOGY/BIOPSY	*AND*	*AND*	*AND*
HBV/HCV MEDICATION	NO	NO	YES

Table S2: Year of diagnosis.

Year of diagnosis	All patients (%)	AH+(%)	AH-(%)
- 2014	2142(20.4)	275(20.1)	1867(20.4)
- 2015	1840(17.5)	302(22.0)	1538(16.8)
- 2016	2430(23.1)	359(26.2)	2071(22.7)
- 2017	2168(20.6)	241(17.6)	1927(21.1)
- 2018	1933(18.4)	194(14.2)	1739(19.0)

Table S3: HCV/HBV Healthcare utilization(HCU)

HCU	Suboptimal care		Optimal care
	Severe	Mild	
Addicted patients N (%)	176(12.8)	401(29.2)	794 (57.9)
Non-addicted patients N (%)	1231(13,5)	3775(41,3)	4136(45,2)
Total patients N(%)	1407(13,4)	4176(39,7)	4930(46,9)

Table S4. A: Sensitivity analysis. Association between opioid addiction and suboptimal healthcare utilization. B: Sensitivity analysis. Association between opioid addiction and severe suboptimal healthcare utilization within suboptimal HCU subgroup.

A						
**Factor**	**OR[a] (univariate)**	**95%CI[a]**	**P value**	**aOR[ab] (multivariate)**	**95%CI**	**P value**
Opioid Addiction	0.61	(0.52, 0.72	<0.0001	0.75	(0.63, 0.89)	0.001
**B**						
**Factor**	**OR[a] (univariate)**	**95%CI[a]**	**P value**	**aOR[ab] (multivariate)**	**95%CI**	**P value**
Opioid Addiction	0.85	(0.63,1.14)	0.27	0.70	(0.51,0.95)	0.020

[a] OR = Odds ratio; CI = confidence interval; aOR = adjusted odds ratio

[b]Adjusted for sex, age, migrant status, comorbidities (see table2)

Table S5. A: Identification of variables contributing to HCU. Association between variables and suboptimal healthcare utilization. B: Identification of variables contributing to HCU. Association between variables and severe suboptimal healthcare utilization within suboptimal HCU subgroup.

A						
Variable	OR[a]	95%CI[a]	P	aOR[a]	95%CI[a]	P
Male sex	0.59	(0.54, 0.63)	<0.0001	0.70	(0.65, 0.76)	<0.0001
Age(y)	0.98	(0.97, 0.98)	<0.0001	0.98	(0.98, 0.98)	<0.0001
Migrant	1.62	(1.50, 1.76)	<0.0001	1.19	(1.09, 1.30)	0.00014
Addiction history	0.60	(0.54, 0.67)	<0.0001	0.73	(0.64, 0.82)	<0.0001
HIV	0.25	(0.21, 0.30)	<0.0001	0.30	(0.25, 0.37)	<0.0001
Cirrhosis	0.29	(0.22, 0.38)	<0.0001	0.38	(0.29, 0.51)	<0.0001
COPD	0.87	(0.67 1.14)	0.32	1.44	(1.09, 1.91)	0.011
Diabetes	0.54	(0.41, 0.73)	<0.0001	0.72	(0.53, 0.97)	0.029
Malignancy	0.57	(0.48, 0.66)	<0.0001	0.77	(0.65, 0.91)	0.0022
**B**						
Covariate	OR[a]	95%CI[a]	P	aOR[a]	95%CI[a]	P
Male sex	1.20	(1.06, 1.36)	0.0038	-	-	-
Age(y)	1.02	(1.02, 1.03)	<0.0001	1.02	(1.01, 1.02)	<0.0001
Migrant	0.64	(0.57, 0.73)	0.00066	0.79	(0.69, 0.91)	0.00066
Addiction history	1.35	(1.12, 1.63)	0.0020	-	-	-
HIV	6.20	(4.31, 8.92)	<0.0001	5.66	(3.91, 8.17)	<0.0001
Cirrhosis	1.25	(0.75, 2.09)	0.394	-	-	-
COPD	1.63	(1.09, 2.43)	0.017	-	-	-
Diabetes	1.06	(0.82, 1.77)	0.822	-	-	-
Malignancy	1.63	(1.26, 2.11)	0.00022	-	-	-

[a] OR = Odds ratio; CI = confidence interval; aOR = adjusted odds ratio.
